# Controllable chaos in hybrid electro-optomechanical systems

**DOI:** 10.1038/srep22705

**Published:** 2016-03-07

**Authors:** Mei Wang, Xin-You Lü, Jin-Yong Ma, Hao Xiong, Liu-Gang Si, Ying Wu

**Affiliations:** 1 School of Physics, Huazhong University of Science and Technology, Wuhan 430074, People’s Republic of China

## Abstract

We investigate the nonlinear dynamics of a hybrid electro-optomechanical system (EOMS) that allows us to realize the controllable opto-mechanical nonlinearity by driving the microwave LC resonator with a tunable electric field. A controllable optical chaos is realized even without changing the optical pumping. The threshold and lifetime of the chaos could be optimized by adjusting the strength, frequency, or phase of the electric field. This study provides a method of manipulating optical chaos with an electric field. It may offer the prospect of exploring the controllable chaos in on-chip optoelectronic devices and its applications in secret communication.

Cavity optomechanics has attracted wide interests in the fields of quantum optics and nonlinear optics in the past a few years^1^,^2^,^3^,^4^,^5^,^6^,^7^,^8^. It explores the intrinsic radiation-pressure interaction between the optical and mechanical modes in the cavity optomechanical system (OMS). Focusing on the classical domain, the nonlinear opto-mechanical interaction can make the mechanical oscillator enter into the regime of self-induced oscillations^9^,^10^,^11^,^12^,^13^, where the backaction-induced mechanical gain overcomes mechanical loss, under the condition of blue-detuned driving. Increasing the driving strength, chaotic motion emerges both in the optical and mechanical modes^14^,^15^,^16^,^17^,^18^. It is shown that this chaos originally comes from the intrinsic optomechanical nonlinearity and does not need periodic perturbation, external feedback, modulation, or delay. This is useful for generating random numbers^19^ and implementing secret information processing^20^,^21^,^22^. However, to apply the generated chaotic signal into the secret communication scheme, good controllability and design flexibility are necessary^23^,^24^.

Hybrid electro-optomechanical system (EOMS)^25^,^26^,^27^,^28^,^29^,^30^,^31^,^32^,^33^,^34^,^35^,^36^,^37^,^38^,^39^,^40^ contains a mechanical oscillator coupled to both an optical cavity and a microwave LC resonator (see Fig. 1), and offers an alternative platform for controlling the optical signal via an electric field. The mechanical oscillator in the EOMS acts as a quantum or classical interface between the optical and microwave modes. Theoretically, it is possible to realize the electric-controlled optomechanically induced transparency^29^, optical nonlinearity^35^ and quantum state transfer between an optical and microwave modes^30^,^32^,^34^ based on this phonon-interface in the hybrid EOMS. Recently, the hybrid EOMS also has been realized experimentally^41^,^42^,^43^, which will inspire the further investigations regarding its basic physical property and the corresponding applications. A natural question is whether we could realize the electric-controlled optical chaos in the hybrid EOMS by using its phonon-interface, which has potential applications in implementing on-chip secret communication.

Here we propose a method to realize the controllable optical chaos in a hybrid EOMS consisting a mechanical oscillator coupled to both an optical cavity and a microwave LC resonator. The microwave resonator is driven by a tunable electric field, which acts as a control part and is separated from the generation part of the chaotic signal. Comparing with the normal OMS, the opto-mechanical nonlinearity in the hybrid EOMS is controllable without changing the optical pumping. Then the switching between the periodic and chaotic motions of the optical field could be realized by only adjusting the electric driving field. Physically, the opto-mechanical nonlinearity will be changed when one drives the LC resonator under different conditions. This ultimately leads to the fact that the chaotic threshold, degree, and lifetime are controllable with respect to an electric field. This study provides a new avenues of manipulating the chaotic signal in on-chip optoelectronic devices, and could effectively avoid the crosstalk between the control field and the chaotic signal in the single-cavity OMS.

## Results

### Hybrid electro-optomechanical system

**Figure 1:**
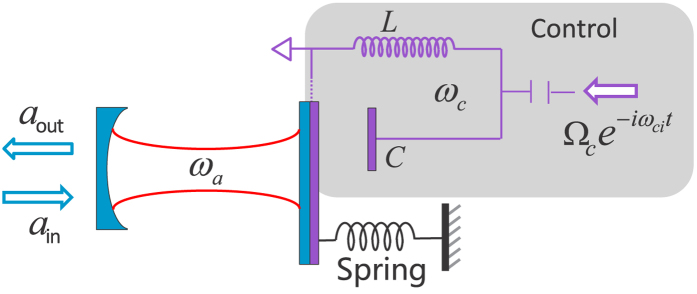
Schematic diagram of the hybrid electro-optomechanical system. The schematic diagram of a hybrid electro-optomechanical system. A mechanical oscillator is parametrically coupled to both an optical cavity and a microwave resonator. The electromechanical subsystem (shaded area) acts as the control port of generating chaos.

We consider a hybrid EOMS depicted in Fig. 1, a mechanical oscillator couples to both an optical cavity and a LC resonator with coupling strengths *ħg*_*a*_ and *ħg*_*c*_. In our proposal, the EOMS is in the weak coupling regime and the values of *g*_*a*_ and *g*_*c*_ have been chosen according to the optomechanical experiment^44^. The microwave resonator (with frequency 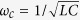) acts as the control port, and is driven by a tunable electric field with amplitude Ω_*c*_ and frequency *ω*_*lc*_. Here Ω_*c*_ is related to the input microwave power *P*_*c*_ and decay rate *κ*_*c*_ by 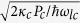. The optical cavity (with frequency *ω*_*a*_) acts as the generation port of chaotic signal, and is pumped by a fixed laser with amplitude Ω_*a*_ and frequency *ω*_*la*_. Here Ω_*a*_ is related to the input optical power *P*_*a*_ and decay rate *κ*_*a*_ by 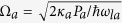. In a frame rotating with frequencies *ω*_*la*_, *ω*_*lc*_, the Hamiltonian for this hybrid system reads


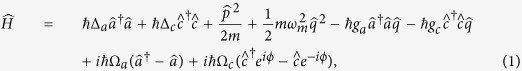


where 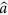 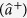, 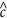 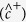 are the corresponding annihilation (creation) operators of the optical and microwave fields, and Δ_*a*_ = *ω*_*a*_ − *ω*_*la*_ (Δ_*c*_ = *ω*_*c*_ − *ω*_*lc*_) is the frequency detuning between the optical cavity (microwave resonator) and the pumping laser (driving electric field). We also have used 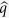, 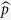 to denote the displacement and momentum of the mechanical oscillator. Here the relative phase *ϕ* between the control and pumping fields is retained since the chaotic motion is usually sensitive to the initial conditions of system.

Generally, the optomechanical interaction in Eq. (1) (i.e., 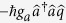) can lead to the chaotic motions of the optical and mechanical modes at a very high-power laser-pumping (~10 milliwatt) in the normal single-cavity OMS^14^. However, here we will demonstrate the generation of the optical chaos in a “weak” pumping regime, i.e., the pumping power *P*_*a*_ = 0.5 mW, which is well below the threshold of chaos in an single-cavity OMS for almost 2 order. Moreover, in our proposal, the mechanical oscillator in the hybrid EOMS couples to both an optical cavity and a microwave resonator. This effectively separates the control and generation ports of the chaotic signal, and increases the controllability of the chaos generation. To present this controllability, in the following sections, we explore the nonlinear dynamics of system by numerically calculating Eqs (2, 3, 4, 5, 6, 7, 8, 9, 10, 11, 12, 13) of the Methods. Note that here the electric driving field is tunable and the optical pumping field is fixed. We define 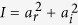 as the intensity of the optical cavity, whose spectrum *S*(*ω*) can be got by using fast Fourier transform.

### Dependence of system dynamics on control-field-power

**Figure 2:**
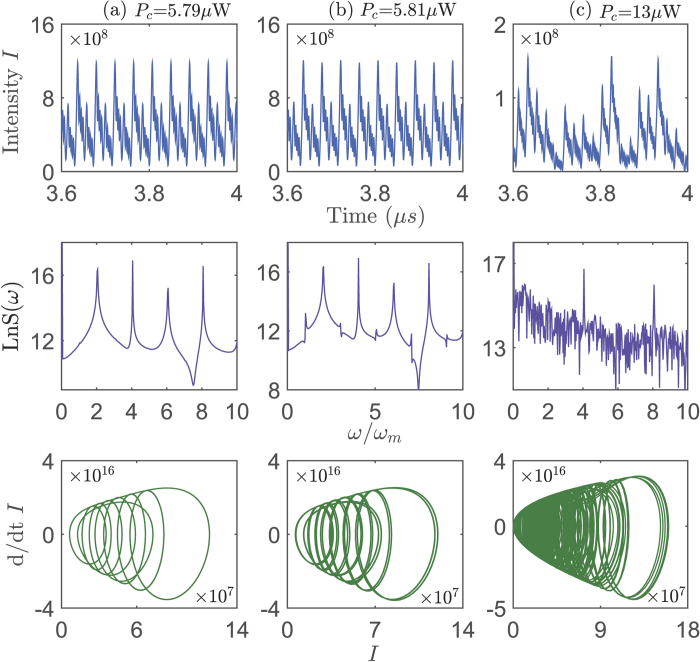
The system dynamics controlled by the driving field. The intracavity field intensity *I* versus time *t*, the power spectrum LnS(*ω*) versus *ω*/*ω*_*m*_, and the optical trajectory in the phase space (the first derivation of *I* versus *I*) for different electric-control-field powers (**a**) *P*_*c*_ = 5.79 *μ*W, (**b**) *P*_*c*_ = 5.81 *μ*W, (**c**) *P*_*c*_ = 13 *μ*W. Here a fixed time interval 0 → 1/3 *μ*s is chosen, and the system parameters are *P*_*a*_ = 0.5 mW, *g*_*a*_ = *g*_*c*_ = 5.59 GHz/nm, *ω*_*m*_ = 73.5 MHz, *ω*_*c*_ = 1.93 GHz, *ω*_*a*_ = 100 THz, Δ_*a*_/*ω*_*m*_ = 1, Δ_*c*_ = 0, *ϕ* = 0, *κ*_*a*_/*ω*_*m*_ = 0.4, *κ*_*c*_/*ω*_*m*_ = 0.8.

First of all, in Fig. 2, we present the evolutions of the intensity *I*, the power spectrum LnS(*ω*) of the intracavity field as well as the optical trajectories in phase space (i.e., the first derivation of *I* versus *I*) under different powers of the control field. It shows that the system dynamics experiences regular to chaotic behaviors when one increases the power *P*_*c*_ of the control field from 5.79 *μ*W to 13 *μ*W. The power spectrum of *I* goes through period, period doubling to a continuum as increasing *P*_*c*_, which characterizes the route to chaos. Accordingly, the optical trajectories in phase space are limited into the regular circles with periodically varying radius under the condition of weak driving, and they become more and more complicated in the strong-driving-regime. The above results show that the generation of the optical chaos can be controlled by adjusting the power of an electric-control-field, which does not interact with the optical cavity.

**Figure 3:**
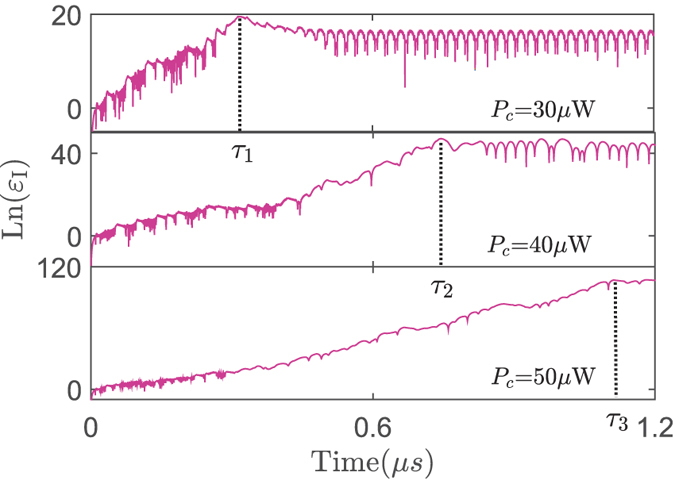
The dependence of the perturbation evolution on the driving strength. The evolution of the intensity perturbation Ln(*ε*_*I*_) for different values of *P*_*c*_. Here *τ*_1_, *τ*_2_ and *τ*_3_ indicate the last times of the chaotic motion. The system parameters are same as those in Fig. 2.

To further explore the influence of *P*_*c*_ on the chaotic dynamics, we present the evolution of a nearby point of *I* (i.e., *I* + *ε*_*I*_ shown in Methods) in Fig. 3. The calculated exponential variation of *ε*_*I*_ indicates how the states of intracavity field vary in temporal domain and phase space. Specifically, the decrease of Ln(*ε*_*I*_) over time indicates that all the nearby points of *I* in phase space will finally oscillate in the limited circles. The flat evolution of Ln(*ε*_*I*_) implies the period-doubling bifurcation. The exponential divergence of Ln(*ε*_*I*_) corresponds to the chaotic dynamics, which reveals that the chaotic regime is extremely sensitive to initial conditions. Figure 3 presents that enhancing the driving power could increase the lifetimes of chaos, which is denoted by *τ*_1_, *τ*_2_, *τ*_3_ and defined by the last time of the chaotic motion, and the degree of chaos corresponding the gradients of Ln(*ε*_*I*_).

### Dependence of system dynamics on driving frequency

**Figure 4:**
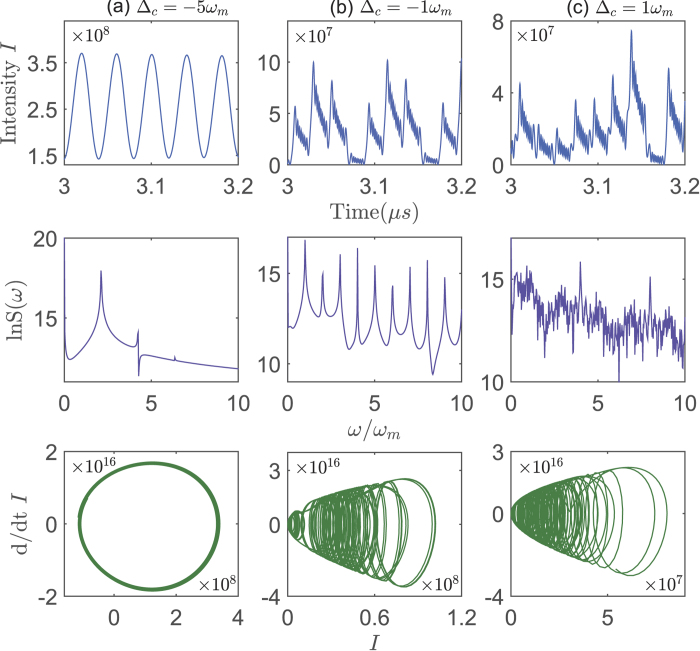
The system dynamics controlled by the frequency detuning. The intracavity field intensity *I* versus time *t*, the power spectrum LnS(*ω*) versus *ω*/*ω*_*m*_, and the optical trajectory in the phase space (the first derivation of *I* versus *I*) for different frequency detuning (**a**) Δ_*c*_/*ω*_*m*_ = −5, (**b**) Δ_*c*_/*ω*_*m*_ = −1, (**c**) Δ_*c*_/*ω*_*m*_ = 1. Here the driven powers *P*_*c*_ = 20 *μ*W, *P*_*a*_ = 0.5 *m*W are fixed, and the other system parameters are same as those in Fig. 2.

**Figure 5:**
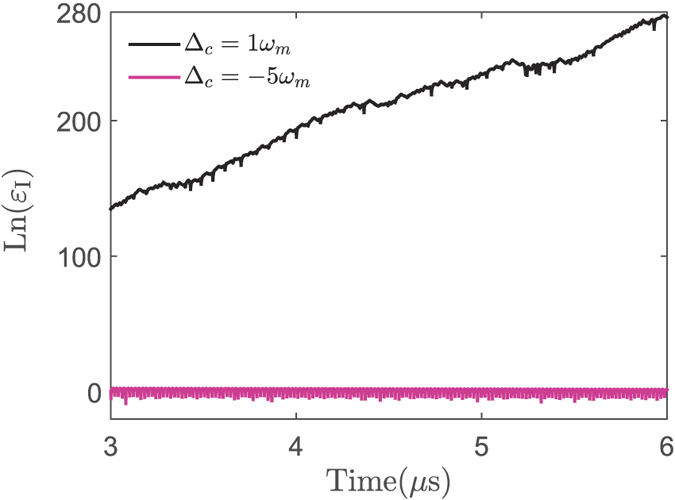
The dependence of the perturbation evolution on the frequency detuning. The evolution of Ln(*ε*_*I*_) for different values of Δ_*c*_. The system parameters are same as those in Fig. 4.

In our proposal, another tunable system parameter is the frequency *ω*_*lc*_ of the electric driving field. In Fig. 4, we present the influence of the frequency detuning Δ_*c*_ (Δ_*c*_ = *ω*_*c*_ − *ω*_*lc*_) on the system dynamics, characterized by the evolutions of *I*, the power spectrum LnS(*ω*), and the optical trajectories in phase space. It shows that the optical trajectory changes to chaotic motion from periodic motion going by the period-doubling when we adjust Δ_*c*_ from blue-detuning to red-detuning. In other words, the red-detuning favors the chaotic dynamics, which is different from the case in the single-mode OMS. Physically, here the optical chaos originally comes from the opto-mechanical nonlinearity, which is decided by the excitation of the mechanical oscillator. The mechanical oscillator is easily excited when the electromechanical subsystem is driven under the condition of red-detuning. Moreover, in Fig. 5, we also present the evolution of a nearby point (*I* + *ε*_*I*_) of *I* under the condition of blue- and red-detuning driving. The exponential divergence of Ln(*ε*_*I*_) under the condition of red-detuning displays the same conclusion. The red-detuning driving could enhance the opto-mechanical nonlinearity and lead to the generation of chaos with the lower optical threshold.

### Dependence of system dynamics on relative phase

**Figure 6:**
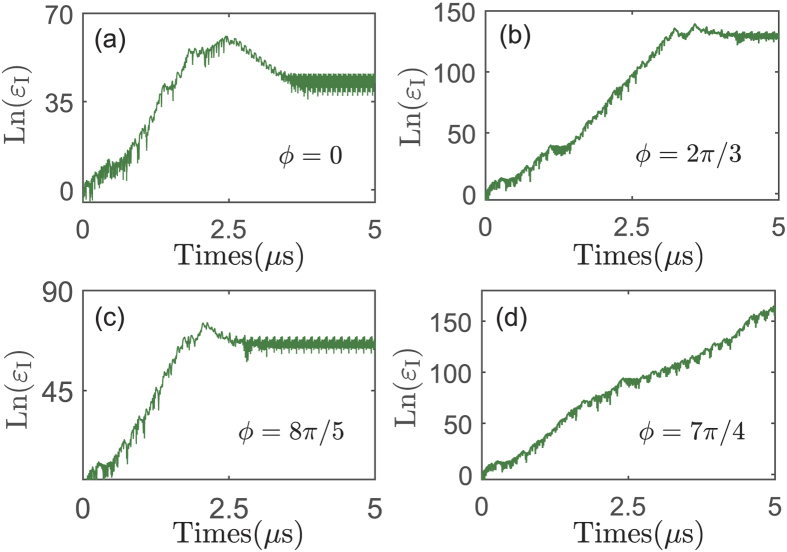
The system dynamics controlled by the relative phase. The evolution of Ln(*ε*_*I*_) for different values of *ϕ* (*ϕ* = *ϕ*_*c*_ − *ϕ*_*a*_). Here *τ*_*j*_ (*j* = 1 − 4) indicates the last time of the chaotic motion. The system parameters are same as those in Fig. 2 except for *P*_*c*_ = 20 *m*W.

Now let’s discuss the influence of the relative phase *ϕ* on the system dynamics (see Fig. 6). Here the relative phase is defined as *ϕ* = *ϕ*_*c*_ − *ϕ*_*a*_ and *ϕ*_*c*_ (*ϕ*_*a*_) is the phase of the electric driving (optical pumping) field. It is shown that the chaotic lifetime (denoted by *τ*_*j*_) is periodically changed when *ϕ* is tuned in the range of 0 to 2*π*. For example the lifetime of chaos increases from *ϕ* = 0 to *ϕ* = 2*π*/3. However it decreases from *ϕ* = 2*π*/3 to 8*π*/5. This result originally comes from the periodic dependence of the opto-mechanical nonlinearity on the relative phase *ϕ*. Such feature provides a new route to manipulate the chaotic signal.

**Figure 7:**
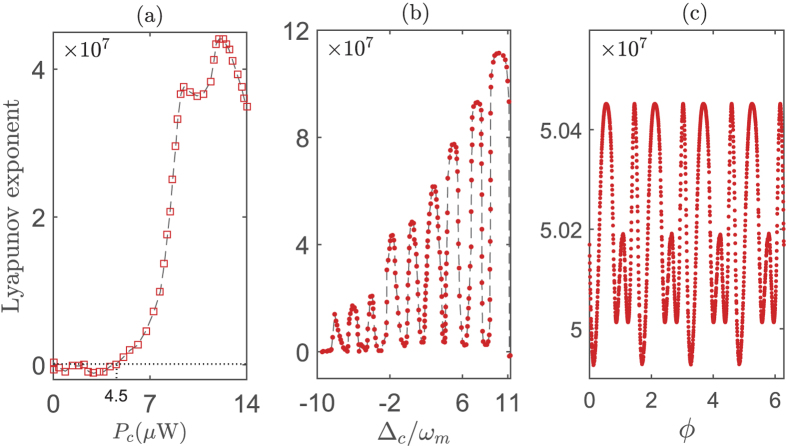
The dependence of Lyapunov exponent on three factors. Lyapunov exponent versus (**a**) *P*_*c*_, (**b**) Δ_*c*_/*ω*_*m*_, and (**c**) *ϕ*. The dotted line indicates the value of *P*_*c*_ corresponding to Lyapunov exponent is equal to zero. The system parameters are same as those in Fig. 2.

Before finishing this section, to illustrate the influences of system parameters on the chaotic dynamics more clearly, we present the dependence of the Lyapunov exponents on the driving power *P*_*c*_, the frequency detuning Δ_*c*_ and the relative phase *ϕ* in Fig. 7. Here the Lyapunov exponent is defined by the logarithmic slope of the perturbation *ε*_*I*_ versus time *t*, and characterizes the separation of trajectories for the identical systems with infinitesimally close initial condition. The negative (positive) value of Lyapunov exponent indicates that the dynamics of system is periodical (chaotic). The dynamical evolution of system exhibits a period doubling behavior when the value of Lyapunov exponent is equal to zero. In Fig. 7(a), the Lyapunov exponent increases (from negative to positive) with enhancing the strength of the control field. This clearly shows the emergence of chaos under the condition of strong electric driving (*P*_*c*_ > 4.5 *μ*W), and it is consistent with the numerical results in Fig. 2. However, Fig. 7(b) indicates that the influence of frequency detuning Δ_*c*_ on the optical chaos is not so simple as exhibited in Fig. 4. The optical chaos emerges periodically with increasing Δ_*c*_. This periodic behavior can also be found in the dependence of chaos on *ϕ*, i.e., Fig. 7(c). Physically, in the hybrid EOMS, the mechanical displacement amplitude *q* is dependent periodically on the frequency (corresponding to Δ_*c*_) and phase (corresponding to *ϕ*) of the electric driving-field. It corresponds to a periodical enhancement of the opto-mechanical nonlinearity, which ultimately leads to the fact that the chaotic degree dependents periodically on Δ_*c*_ and *ϕ*.

**Figure 8:**
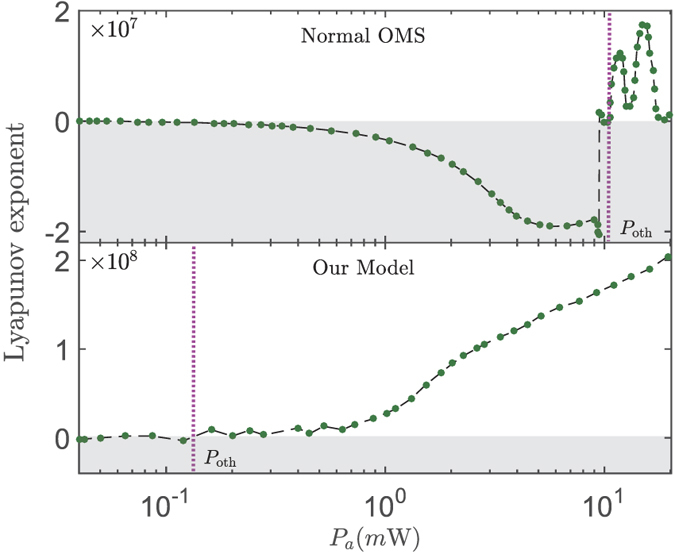
The chaotic threshold in two different models. Lyapunov exponent versus optical pump-power *P*_*a*_. Here *P*_oth_ denotes the optical threshold of chaos, and the shaded regions correspond to Lyapunov exponent is less than zero. The system parameters are same as those in Fig. 2 except for *P*_*c*_ = 10 *μ*W.

To explore the role of the optical pump laser, in Fig. 8, we present the dependence of Lyapunov exponent on *P*_*a*_ in the normal OMS and our model, respectively. It clearly shows that the chaotic motion emerges (Lyapunov exponent > 0) when the pumping power *P*_*a*_ > *P*_oth_ (*P*_oth_ is defined by the optical pumping power corresponding to the Lyapunov exponent equal to zero). More interestingly, comparing with the normal OMS, the optical threshold *P*_oth_ of chaos is reduced almost two order in our proposal. Physically, this is due to the opto-mechanical nonlinearity is enhanced when the electricmechanical subsystem is driven with an electric field. This result provides an alternative method to decrease the chaotic threshold in the OMS. Lastly, it should be noted that, in Figs 7 and 8, a non-zero optical (or electrical) driving power has been chosen, i.e., *P*_*a*_ = 0.5 mW (or *P*_*c*_ = 10 *μ*W), which induces a considerable opto-mechanical nonlinearity even without the electrical (or optical) driving-field. This retained opto-mechanical nonlinearity leads to the result that the dynamics of system enters easily into the chaotic regime, and then the Lyapunov exponents are almost non-negative in the chosen parameter range of Figs 7 and 8.

## Discussion

We have presented a practical method to achieve the controllable optical chaos in a hybrid EOMS. Via investigating the nonlinear dynamics of system, we shown that the optical chaos could be switched on and off by driving the electromechanical subsystem with a tunable electric field. This is due to that the opto-mechanical nonlinearity (the essence of the generation of chaos) can be controlled by an electric field in the hybrid EOMS. We analyzed in detail the influences of the frequency, strength and phase of the electric control field on the chaotic dynamics, including the degree and the lifetime of chaos. Moreover, we also shown that the optical threshold of chaos is reduced dramatically in our model due to the enhanced opto-mechanical nonlinearity when the electromechanical subsystem is driven. This study provides a promising route for controlling the optical nonlinear dynamics, especially the generation of the optical chaos, with an electric field, and has potential applications in implementing secret communication on the integrated chips.

## Methods

To explore the nonlinear dynamics of system, we employ the semiclassical equations of motion (setting 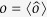, 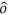 is any optical or mechanical operator)


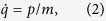



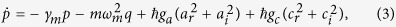



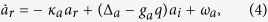



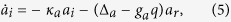



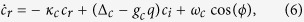



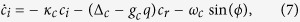


where *γ*_*m*_ is the damping rate of the mechanical oscillator and we have defined *a* = *a*_*r*_ + *ia*_*i*_, *c* = *c*_*r*_ + *ic*_*i*_ (*a*_*r*_, *a*_*i*_, *c*_*r*_, *c*_*i*_ are real numbers) for simplifying the discussion of the chaotic property of system. Therefore, the Eqs (2, 3, 4, 5, 6, 7) are given by splitting the real and imaginary parts into different equations. Here the quantum correlations of photon-phonon have been safely ignored in the semiclassical approximation, which is valid in the concerned weak-coupling regime^14^. Eqs (2, 3, 4, 5, 6, 7) show that the intracavity field intensities and the mechanical deformation influence each other during the system evolution via the optomechanical interaction. Specifically, Eqs (2) and (3) describe the motion of mechanical oscillator, and Eqs (4, 5, 6, 7) describe the dynamics of the optical and microwave modes. The quadratic terms 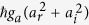 and 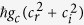 in Eq. (3) and the mixed terms −*g*_*a*_*qa*_*i*_, *g*_*a*_*qa*_*r*_, and −*g*_*c*_*qc*_*i*_, *g*_*c*_*qc*_*r*_ in Eqs (4, 5, 6, 7) clearly present the nonlinear interaction between the optical (or microwave) and the mechanical modes.

To calculate the Lyapunov exponent of the dynamical evolution, we linearize Eqs (2, 3, 4, 5, 6, 7) and introduce the evolution of a perturbation 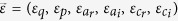,


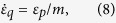



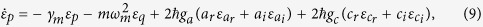



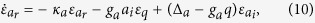



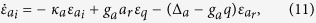



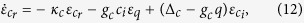



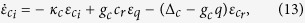


which characterizes the divergence of nearby trajectories in phase space. Here we define 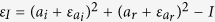 and use its logarithm Ln(*ε*_*I*_) to show the tendency of the perturbation. Then the logarithmic slope of the perturbation *ε*_*I*_ versus time *t* is defined as Lyapunov exponent, the negative and positive values of the Lyapunov exponent, respectively denotes states of the dynamical system, being out of and in the chaotic motions.

## Additional Information

**How to cite this article**: Wang, M. *et al*. Controllable chaos in hybrid electro-optomechanical systems. *Sci. Rep.* **6**, 22705; doi: 10.1038/srep22705 (2016).
